# Estimation of seed yield in oilseed rape to identify the potential of semi-resynthesized parents for the development of new hybrid cultivars

**DOI:** 10.1371/journal.pone.0215661

**Published:** 2019-04-18

**Authors:** Laurencja Szała, Zygmunt Kaczmarek, Wiesława Popławska, Alina Liersch, Marek Wójtowicz, Marcin Matuszczak, Zdzisław R. Biliński, Katarzyna Sosnowska, Michał Stefanowicz, Teresa Cegielska-Taras

**Affiliations:** 1 Plant Breeding and Acclimatization Institute—National Research Institute, Department of Genetics and Breeding of Oilseed Crops, Poznań, Poland; 2 Institute of Plant Genetics, Polish Academy of Sciences, Department of Biometry and Bioinformatics, Poznań, Poland; 3 Plant Breeding Company Smolice Ltd., Group IHAR, Division Bąków, Poland; 4 Plant Breeding Company Strzelce Ltd., Group IHAR, Division Małyszyn, Poland; Huazhong University of Science and Technology, CHINA

## Abstract

Resynthesized (RS) *Brassica napus* can be used to increase the genetic diversity of this important crop plant and to develop the heterotic gene pool required for successful hybrid breeding programmes. The level of heterosis in F_1_ hybrids depends on the individual performance of the parents and on the degree of genetic difference between them. However, RS forms obtained from crosses of *B*. *rapa* ssp. with *B*. *oleracea* ssp. possess many undesirable agronomic traits, such as low quality of seeds, low yield and seed oil content, high erucic acid level in the oil and high glucosinolate content in seed meal. Therefore, RS oilseed rape needs to be improved by crossing with natural double-low oilseed rape, leading to selected double-low quality semi-RS lines that can be used for breeding. In this study, we evaluated the seed yield potential of F_1_ hybrids derived from crosses between Ogura cytoplasmic male-sterility (CMS) lines and doubled haploid (DH) semi-RS lines with restorer gene in three locations in Poland. The genotype by environment interaction (GE interaction) and general combining ability (GCA) of the restorer and CMS line effects, as well as the effects of heterosis, were also assessed. The results of the study provide the first insights into the use of semi-RS lines as components for the development of new hybrid cultivars. Even the introduction of 50% of the RS oilseed rape genotype to natural restorer lines resulted in a marked heterosis effect, with seed yield ranging from 4.56% to 90.17% more than that of the better parent. The yield of the best hybrid amounted to 108.6% of the seed yield of the open-pollinated cultivar Monolit and 94.4% of that of the hybrid cultivar Arsenal. The best DH semi-RS line S1, which had a significantly positive GCA for seed yield, can be recommended as a possible parent for inclusion in breeding programmes aimed at developing new hybrid cultivars.

## Introduction

Oilseed rape (*Brassica napus* L.) is considered a relatively young species. Although its origin is not yet fully explained, it is thought that *B*. *napus* was formed as a result of multiple spontaneous and independent hybridizations of *B*. *rapa* and *B*. *oleracea* on the coast of northern Europe, where both diploid parental species grow wild. Other researchers believe that oilseed rape originated in the Mediterranean region or in western or northern Europe [1]. It is also possible that *B*. *napus* could have formed elsewhere from crosses between different forms of *B*. *oleracea* and *B*. *rapa* [2]. Based on the sequencing of its polyploid genome, Chalhoub et al. have suggested that the hybridization that gave rise to *B*. *napus* occurred about 7500 years ago [3].

Oilseed rape began to be used in Europe as a cultivated plant 500 years ago, but it was not until the nineteenth century that it was grown widely. However, since the 1960s, strong selection pressure has been applied to breed zero erucic acid and low glucosinolate content in seeds (double-low quality), which, together with globalization in recent years, has led to a narrowing of the gene pool in this species. This relatively limited genetic diversity of modern oilseed rape breeding materials restricts the potential for further improvement in yield [4]. In contrast, *B*. *rapa* and *B*. *oleracea* are both highly polymorphic and offer a much broader genetic variability, and this can be exploited for oilseed rape improvement via experimental hybridization–resynthesis–from diploid progenitors. Resythesized (RS) *B*. *napus* can be used to increase genetic diversity and to develop a heterotic gene pool, which is one requirement for successful hybrid breeding programmes [5]. The number and diversity of available Brassica gene bank accessions is enormous. Boukema and van Hintum report that there are 20,106 accessions of *B*. *oleracea* and 18,224 accessions of *B*. *rapa* [6]. These high numbers of accessions in both progenitor species represent enormous potential for creating new variation in *B*. *napus*. However, to date, resynthesized forms obtained from crosses of *B*. *rapa* ssp. with *B*. *oleracea* ssp. possess many undesirable agronomic traits such as low seed quality, with low yield and seed oil content, high erucic acid level in the oil and high glucosinolate content in seed meal [5,7–8]. Moreover, like both ancestral species, RS *B*. *napus* is self-incompatible [9]. In contrast, natural amphidiploid *B*. *napus* is self-compatible. Therefore, resynthesized oilseed rape needs to be improved by crossing with natural double-low oilseed rape and selection of double-low semi-RS lines, and only then should it be introduced into breeding programmes.

It has long been known that Brassicaceae crops show strong heterosis. Across the whole Brassica group, oil seed crops show hybrid vigor in terms of seed yield, giving 35–100% higher [10–11] and even up to 200% higher of the yield of the parental lines [12–13]. Many cytoplasmic male-sterility (CMS) systems for hybrid breeding to exploit heterosis phenomenon have been elaborated in cultivated Brassica species. One of the most effective and commonly used systems for the control of cross-pollination in oilseed rape is Ogura cytoplasmic male-sterility (CMS), which is characterized by a sterile male cytoplasm and the *Rfo* restorer gene [14]. Generally, the level of heterosis in F_1_ hybrids depends on the individual performance of parents and on the degree of genetic difference between them. The introduction of artificially resynthesized oilseed rape to the restorer lines and the development of semi-RS lines with the *Rfo* gene provide an opportunity to establish a new gene pool and novel *B*. *napus* varieties that are different to modern breeding materials.

For several years, we have been developing restorer lines with resynthesized oilseed rape for basic studies and breeding purposes. The breeding materials have been evaluated in experimental field conditions [15]. One objective of the current study was to evaluate the seed yield potential of F_1_ test hybrids derived from crosses between Ogura CMS lines and semi-RS doubled haploid (DH) lines with *Rfo* gene in three locations in Poland. The genotype by environment interaction (GE interaction), general combining ability (GCA) of the restorer and CMS lines, and the effect of heterosis were also evaluated.

## Materials and methods

### Plant material

The material consisted of fifteen hybrids (M1S1, M2S1, M3S1, M4S1, M5S1, M1S3, M2S3, M3S3, M4S3, M5S3, M1S4, M2S4, M3S4, M4S4, M5S4) and their parental lines: five Ogura CMS lines (CMS1, CMS2, CMS3, CMS4, CMS5) and three semi-RS DH lines with the *Rfo* gene as restorer lines (S1, S3, S4). The development and selection of semi-RS restorer lines were described in the previous paper by Szała et al. [15]. Hybrid seeds from crosses between male sterile CMS lines and semi-RS restorer lines were produced in isolation tents under field conditions. Two winter oilseed rape cultivars served as a control: hybrid cultivar Arsenal F_1_ and open-pollinated cultivar Monolit.

### Experimental conditions

**Fig 1:**
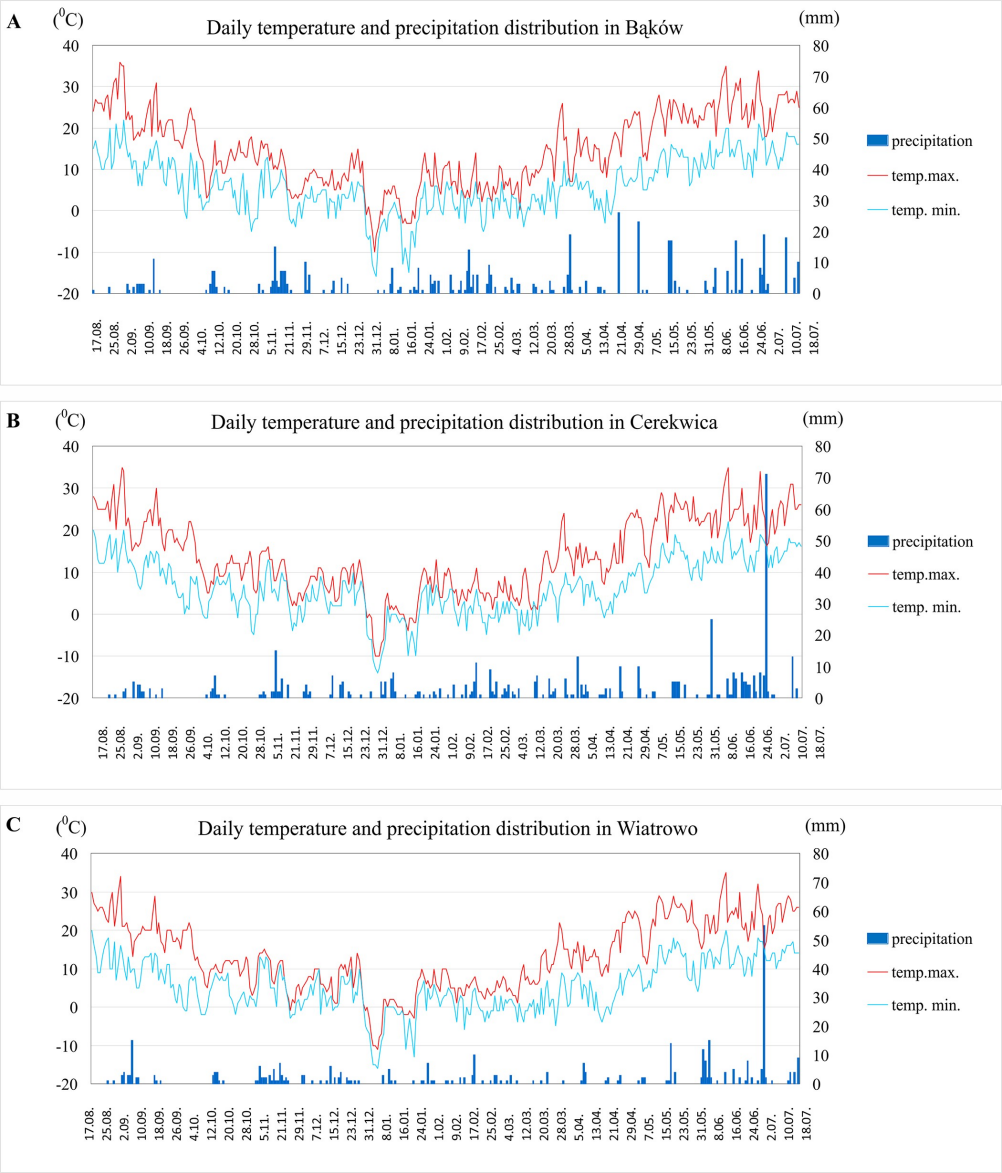
Weather conditions in Bąków, Cerekwica and Wiatrowo in 2015/2016.

Field trials were carried out in the 2015/16 season in three locations: Cerekwica (CER - 52°31′16″ N; 16°41′30″ E), Bąków (BAK—50°57′44″ N; 18°18′45″ E) and Wiatrowo (WIA - 52°45′15″ N; 17°08′19″ E) on sandy clay soil. The experiments including 25 genotypes were conducted in a randomized complete block design with three replications and a plot size of 10 m^2^. Oilseed rape genotypes were sown on 1^st^ September 2015 at Cerekwica, 27^th^ August at Bąków and 28^th^ August 2015 at Wiatrowo with a sowing density of 80 seeds/m^2^ and harvested 25^th^ July, 19^th^ July and 19^th^ July, respectively. Full chemical protection of plants was applied. Other agronomic practices were similar to those used in commercial planting in the area. The weather conditions in Poland in the 2015/16 season were unfavorable; the lack of snow cover and very cold temperatures at the beginning of January 2016 caused severe damage to the plants. Additionally, heavy rain in July delayed the harvest and reduced yields (Fig 1).

### Statistical analysis

As a first step, one-way analysis of variance (ANOVA) was performed for seed yield to determine differences among the studied genotypes for each of the three field experiments, according to Gomez and Gomez [16]. In the next step, particularly analysis of genotype by environmental interaction was performed. Additionally, the analysis of general combining ability (GCA) for seed yield of Ogura CMS lines and semi-RS DH lines was carried out. The data from all three experiments were processed using the computer program SERGEN [17] based on the methods developed by Kaczmarek [18] and Caliński et al. [19, 20, 21]. Heterosis of hybrids between Ogura CMS lines and semi-RS DH lines with the *Rfo* gene was calculated in respect of the mean seed yield value of both parents and the mean of the better parent. All data are available at: http://dx.doi.org/10.17504/protocols.io.x9wfr7e

## Results

Analysis of the seed yield of fifteen F_1_ hybrids, three semi-RS DH lines with restorer gene, five Ogura CMS lines and two cultivars, Arsenal and Monolit, as controls was carried out on the results obtained from three locations. The mean values of seed yield and the degree of heterosis over mid-parent and the better parent are presented in Table 1. The highest mean yield for all genotypes was obtained in Wiatrowo. The mean seed yield over all locations was 20.06 dt·ha^-1^ and in individual genotypes (hybrids and their components) ranged from 14.03 to 27.46 dt·ha^-1^. The low yields in the season in question were a result of unfavorable weather conditions. For comparison, an year earlier (2014/2015) at COBORU (Research Center for Cultivar Testing) experimental stations located in south-western Poland, where Bąków is located, cv. Monolite and Arsenal yielded 39.3 and 47.1 dt·ha^-1^, respectively and two years earlier (2013/14)– 50.9 and 59.6 dt·ha^-1^, respectively. Whereas, at COBORU experimental stations located in the region of Poland, where Cerekwica and Wiatrowo are located, the Monolit and Arsenal cultivars yielded 39.3 and 43.8 dt·ha^-1^in the season 2014/15 and 49.4 and 50.9 dt·ha^-1^in the season 2013/2014, respectively [22].

**Table 1:** Mean seed yield values (dt·ha^-1^) of F_1_ hybrids, parental lines and control varieties obtained in three environments.

No. of genotype	Genotype	Environment			Mean	Heterosis over	
		Bąków	Cerekwica	Wiatrowo		mid-parent (%)	better parent (%)
1	M1S1	20.47	22.60	22.07	21.71	53.21	51.71
2	M2S1	24.27	18.53	28.27	23.69	50.79	36.23
3	M3S1	21.17	22.07	24.80	22.68	47.80	36.13
4	M4S1	23.47	19.67	27.80	23.65	59.73	51.83
5	M5S1	25.43	21.83	22.97	23.41	64.86	62.91
6	M1S3	19.07	14.57	19.87	17.84	24.03	23.48
7	M2S3	18.40	25.60	23.50	22.50	41.38	29.38
8	M3S3	16.97	17.00	24.60	19.52	25.53	17.17
9	M4S3	21.83	18.70	29.93	23.49	56.55	50.87
10	M5S3	24.87	32.43	25.07	27.46	90.63	90.17
11	M1S4	19.57	18.57	22.93	20.36	36.51	31.18
12	M2S4	18.03	18.20	22.97	19.73	19.90	13.46
13	M3S4	18.03	11.03	23.20	17.42	10.13	4.56
14	M4S4	17.40	17.00	18.37	17.59	13.15	12.97
15	M5S4	17.57	17.23	21.93	18.91	26.53	21.84
16	S1	12.87	8.80	20.43	14.03		
17	S3	11.90	14.77	15.67	14.11		
18	S4	10.43	9.47	26.67	15.52		
19	CMS1	14.73	12.60	15.60	14.31		
20	CMS2	12.30	17.80	22.07	17.39		
21	CMS3	14.90	20.77	14.30	16.66		
22	CMS4	14.63	12.60	19.47	15.57		
23	CMS5	11.87	12.07	19.17	14.37		
24	cv. Arsenal	26.53	33.87	26.80	29.07		
25	cv. Monolit	25.33	23.83	26.70	25.29		
	Mean	18.48	18.46	22.61	19.85	41.38	35.59

The magnitude of heterosis over the mid-parent and better parent values ranged from 10.13% to 90.63% and from 4.56% to 90.17%, respectively.

Preliminary one-way ANOVA indicated that in Cerekwica and Bąków there were highly significant differences among the studied genotypes. The combined analysis of variance for yield revealed significant effects of environment, genotype and GE interaction. The most important effect was that caused by genotype, which represented 48.77% of the total sum of squares, and this factor was the highest source of variation (Table 2). The sum of squares for environmental and GE effects represented 13.12% and 20.39% of the total variation, respectively.

**Table 2:** Results of overall analysis of variance for yield of F_1_ hybrids, parental lines and control cultivars.

Source of variation	Degree of freedom	Sum of squares	Mean squares	F-statistic	Variability explained (%)
Environment	2	284.75	145.38	66.12**	10.45
Genotype	24	1329.11	55.38	-	48.77
Genotype × environment	48	555.67	11.58	5.38**	20.39
Regression on environment	24	351.58	14.65	-	
Regression deviation	24	204.09	8.50	3.95**	
Experimental error	144		2.15		

**: significant at 0.01 level

Of the 25 studied genotypes, cultivar Arsenal and hybrid M5S3 demonstrated the highest mean seed yields of 29.07 dt·ha^-1^ and 27.46 dt·ha^-1^, respectively (Table 1). However, in the analysis of GE interaction, the estimates of the main effects of genotype indicated that yield was significantly higher than the overall mean in hybrids M3S1 and M4S1, as well as in cultivar Monolit. There was also a significantly lower seed yield in hybrid M5S4, semi-RS DH line S3 and three Ogura CMS lines, CMS1, CMS4 and CMS5 (Table 3).

**Table 3:** The results of testing the hypotheses concerning evaluation of F_1_ hybrids, parental lines and control cultivars and their interaction with the environment.

No. of genotype	Genotype	Estimate of main effect	F-statistic for		Coefficient of		F-statistic for	
			main effect	interaction	regression	determination (%)	regression	deviations
1	M1S1	1.83	1.90	2.65*	-0.87	79.22	3.81	1.10
2	M2S1	3.84	4.14	5.17**	0.67	23.67	0.31	7.89***
3	M3S1	2.83	46.78**	0.25	-0.23	59.38	1.46	0.20
4	M4S1	3.80	8.57*	2.44*	0.51	29.38	0.42	3.45*
5	M5S1	3.56	3.50	5.26***	-1.16	70.14	2.35	3.14*
6	M1S3	-2.01	2.25	2.62*	-0.26	7.04	0.08	4.87**
7	M2S3	2.65	1.37	7.42***	-0.64	15.33	0.18	12.56***
8	M3S3	-0.33	0.08	1.95	0.84	99.98	4246.81***	0.00
9	M4S3	3.64	3.14	6.11***	1.34	81.09	4.29	2.31
10	M5S3	7.61	5.08	16.54***	-1.87	58.37	1.40	13.77***
11	M1S4	0.51	2.88	0.13	-0.07	9.14	0.10	0.23
12	M2S4	-0.12	0.23	0.09	0.17	95.04	19.16	0.01
13	M3S4	-2.43	0.93	9.22***	1.10	36.36	0.57	11.73***
14	M4S4	-2.26	5.17	1.43	-0.72	98.68	74.94*	0.04
15	M5S4	-0.94	33.83**	0.04	0.10	67.15	2.04	0.02
16	S1	-5.82	7.23	6.80***	1.32	71.10	2.46	3.93**
17	S3	-5.74	31.23**	1.53	-0.44	34.44	0.53	2.01
18	S4	-4.33	1.06	25.65***	3.05	99.63	266.54**	0.19
19	CMS1	-5.54	33.77**	1.32	-0.53	58.72	1.42	1.09
20	CMS2	-2.46	1.75	5.02***	0.69	26.42	0.36	7.39***
21	CMS3	-3.19	1.08	13.67***	-1.86	69.68	2.30	8.29***
22	CMS4	-4.28	27.52**	0.97	0.42	49.74	0.99	0.97
23	CMS5	-5.48	28.64**	1.52	0.74	99.58	237.00**	0.01
24	cv. Arsenal	9.22	7.85	15.70***	-1.83	58.65	1.42	12.98***
25	cv. Monolit	5.44	46.63**	0.92	-0.49	70.76	2.42	0.54

*, **,***: significant at 0.05, 0.01 and 0.001 levels, respectively

None of the studied genotypes with a significantly higher or lower main effect showed any interaction with the environment except hybrid M4S1. In total, nine hybrids, two CMS lines, two semi-RS DH lines and one control cultivar showed significant interaction with the environment. Among these were the two highest yielding genotypes, cultivar Arsenal and hybrid M5S3, which both gave yields higher than the overall mean. Analysis of linear regression allowed the nature of individual genotype interaction with the environment to be determined and identified six hybrids [M1S1 (genotype no. 1), M2S1 (no. 2), M4S1 (no. 4), M1S3 (no. 6), M4S3 (no. 9) and M3S4 (no. 13)], two semi-RS restorer lines [S1 (no. 16) and S4 (no. 18)], and the CMS2 line (no. 20) as intensive genotypes with the highest adaptation to more favorable growing conditions. The remaining, unstable genotypes, i.e. M5S1 (no. 5), M2S3 (no. 7), M5S3 (no. 10), CMS3 (no. 21) and cv. Arsenal (no. 24), showed the best adaptability to less favorable environments (Table 1 and Table 3).

**Fig 2:**
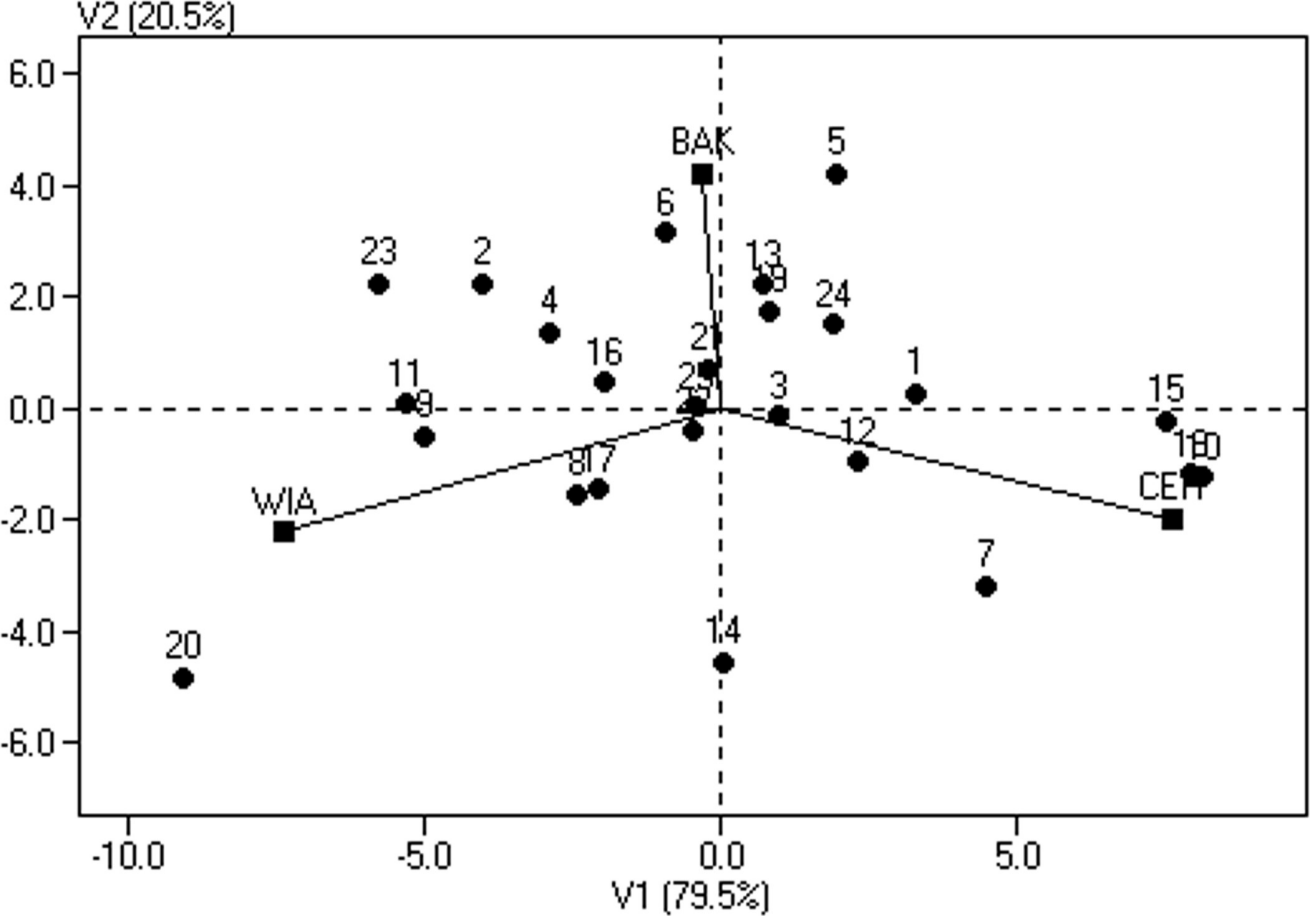
Biplot for estimates of the genotype by environment interaction of genotypes in relation to locations. Genotypes are indicated by points and locations by lines.

A study of the structure of the GE interaction using principal component analysis provided additional information about the behavior of all oilseed rape genotypes in the three environments used (Fig 2). An environment with a high GE interaction is characterized by a long distance from the origin of the coordinate system. The yields of genotypes in such a location differed significantly from the average yields obtained in the experiment. The distance of the points marked as genotypes from the origin of the system is a measure of the interaction size of each of these genotypes with the environment. The most stable genotypes, i.e. M5S4 (no. 15), M2S4 (no. 12), M1S4 (no. 11) and M3S1 (no. 3), are closest to the origin of the system, while unstable genotypes, showing a strong reaction to environmental conditions, are placed far from the origin of the system. These include the following genotypes: S3 (no. 18), M5S3 (no. 10), cv. Arsenal (no. 24) and CMS3 (no. 21). At the same time, the positioning of a genotype near a given location (marked by lines), indicates with which environment this genotype best corresponded.

The results of testing the GCA effects of the CMS lines and their interaction with the environment are given in Table 4. Of the five maternal lines, three (CMS2, CMS4 and CMS5) exhibited positive but non-significant GCA effects and two were characterized by negative GCA effects, including CMS1, which showed a statistically significant negative effect. The GCA effects for seed yield of the CMS1 and CMS2 lines were stable, while the GCA effects of the CMS3, CMS4 and CMS5 lines showed a statistically significant interaction with the environment.

**Table 4:** Testing the GCA effects for seed yield of CMS lines and their interaction with a given environment.

CMS *ogura* line	Average yield of hybrids	Estimation of main effect	F-statistic for		Coefficient of		F-statistic for	
			main effect	interaction	regression	determination (%)	regression	deviations
CMS1	19.97	-1.36	8.69*	1.65	-0.33	85.14	5.73	0.49
CMS2	21.97	0.64	2.30	1.39	0.10	8.58	0.09	2.54
CMS3	19.87	-1.46	2.32	7.06***	0.72	95.38	20.63	0.65
CMS4	21.58	0.24	0.10	4.45**	0.53	77.70	3.49	2.12
CMS5	23.26	1.93	1.98	14.47***	-1.02	93.52	14.43	1.88

*, **, ***: significant at 0.05, 0.01 and 0.001 levels, respectively

**Fig 3:**
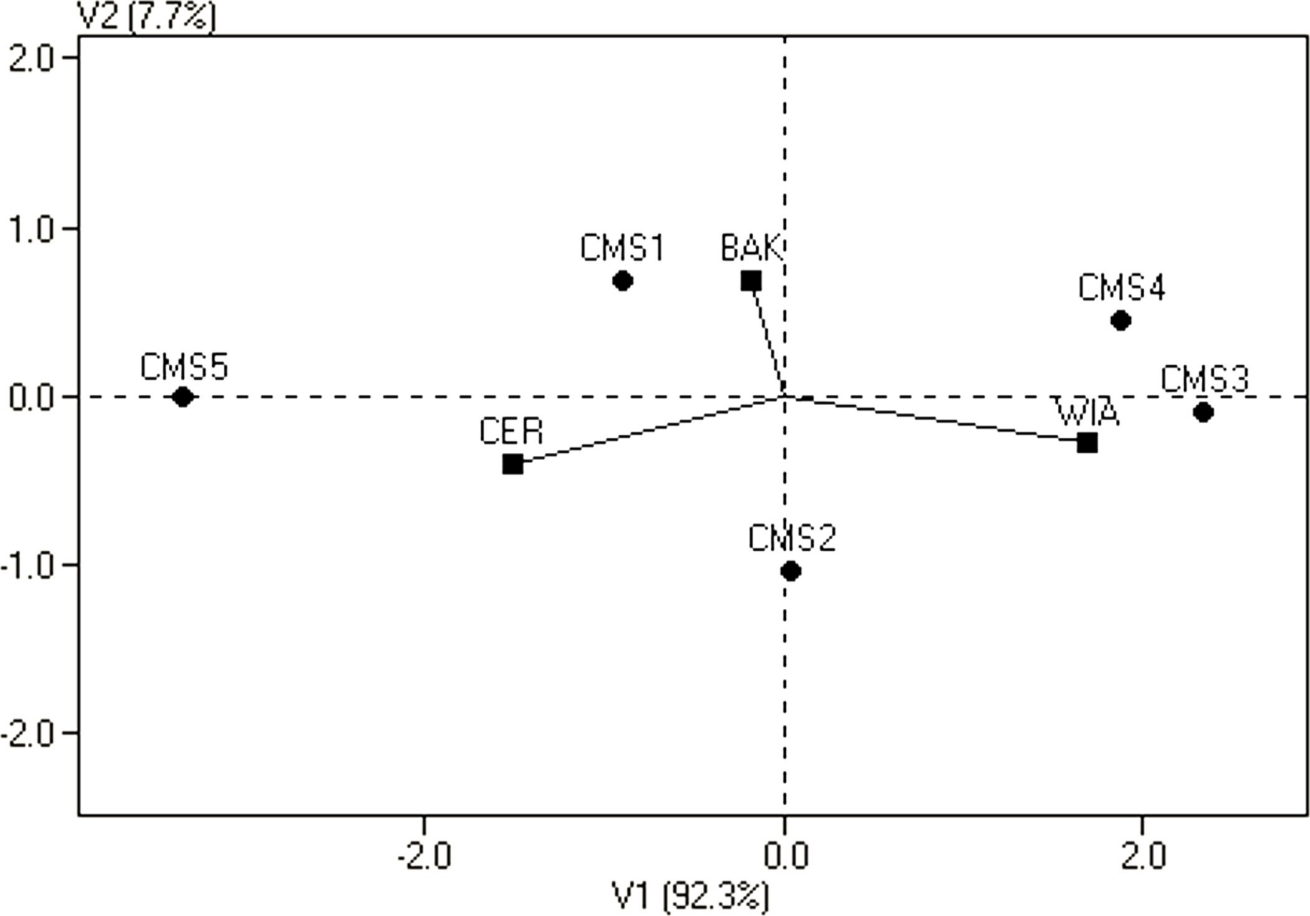
Biplot for estimates of the genotype by environment interaction of GCA of CMS lines in relation to locations. Genotypes are indicated by points and locations by lines.

A graphical configuration of GE interaction for GCA of CMS lines is presented in Fig 3. The CMS1 and CMS2 lines, which show stable GCA effects, are closest to the origin of the principal coordinate axes, while the GCA of the other three lines, which located further from the origin, depended on the environment. The position of the CMS line near a given location indicates in which environment the GCA effects for this line were the best.

**Fig 4:**
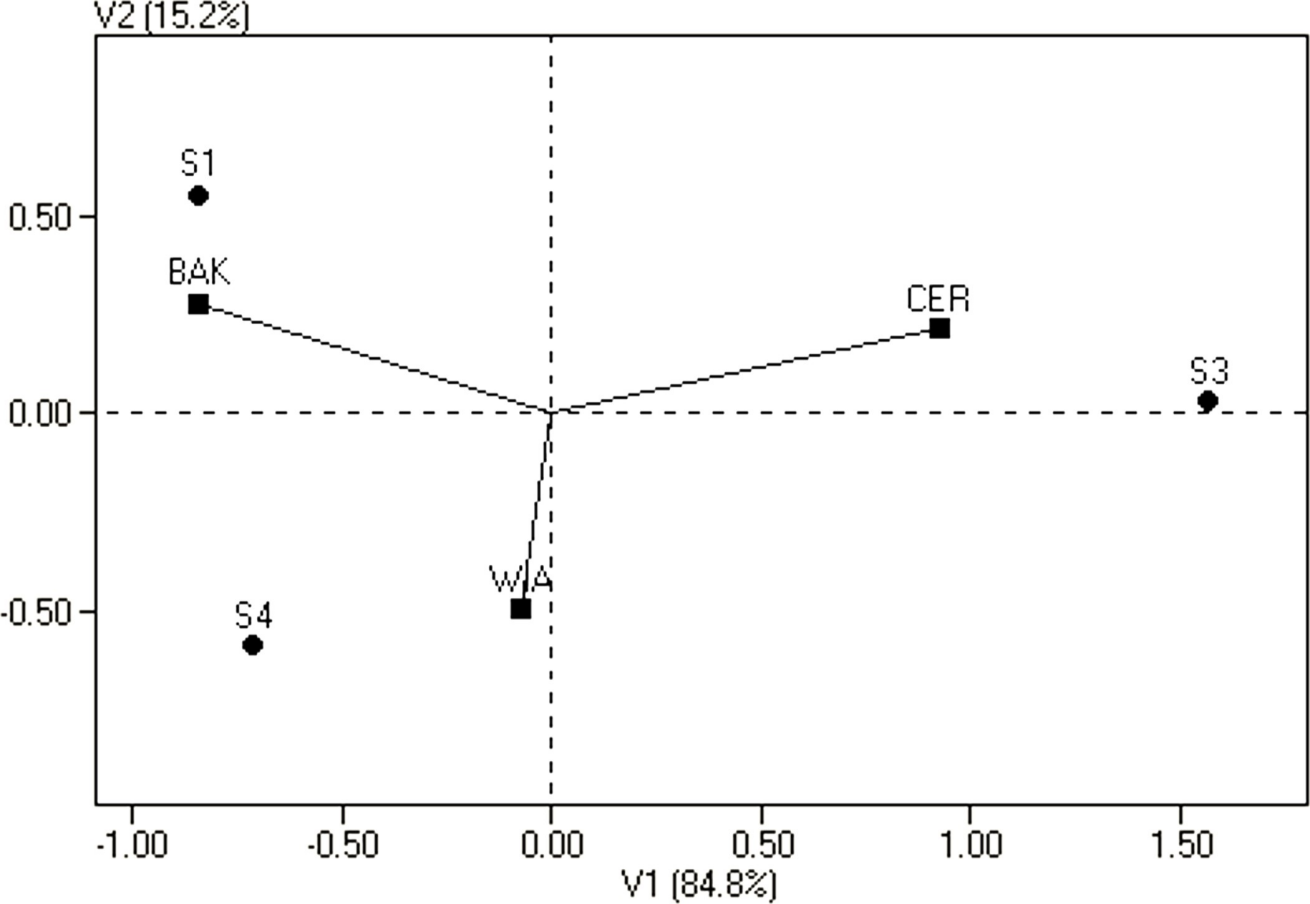
Biplot for estimates of the genotype by environment interaction of GCA of semi-RS lines in relation to locations. Genotypes are indicated by points and locations by lines.

Genetic analysis of hybrids between CMS and restorer lines also allowed the GCA effects of restorers to be obtained. The significance of their main effects as well as the significance of their interactions with the environment were tested (Table 5). Among the restorers, the best general combiner was the semi-RS DH line S1 with a significant (α = 0.05) and positive GCA effect, while the semi-RS DH line S4 expressed high but negative GCA. Of the five hybrids that were the offspring of the S4 restorer, four of them yielded lower than the overall mean (Table 1). The third restorer line S3 demonstrated poor GCA. The GCA of two of the restorer semi-RS lines S1 and S3, depended significantly on the environment. The GCA effects of the restorer semi-RS DH line S4 also depended on the environment, although this interaction was not statistically significant. These results are shown graphically in Fig 4, where all three semi-RS lines are located at a considerable distance from the origin of the principal coordinate axes.

**Table 5:** Testing the GCA effects for seed yield of restorer lines (semi-RS DH lines) and their interaction with a given environment.

Restorer line	Average yield of hybrids	Estimation of main effect	F-statistic for		Coefficient of		F-statistic for	
			main effect	interaction	regression	determination (%)	regression	deviations
S1	23.03	1.70	16.83*	2.64*	-0.10	10.75	0.12	4.72**
S3	22.16	0.83	1.69	6.28***	-0.13	6.98	0.08	11.68***
S4	18.80	-2.53	44.72**	2.20	0.23	64.78	1.84	1.55

*, **, ***: significant at 0.05, 0.01 and 0.001 levels, respectively

Comparing the estimates of the interaction of parental forms with the environment and the estimates of the interaction of their GCA effects with the environment, it can be seen that the GCA effects of stable parental forms were unstable and, vice versa, the GCA effects of unstable parental forms were stable. Only in two cases were these estimates consistent. The stable line, CMS1, revealed stable GCA effects and the semi-RS DH line S1, which displayed interaction with the environment, showed unstable GCA effects.

## Discussion

Resynthesized *B*. *napus* has gained much interest for its high genetic diversity compared to natural oilseed rape cultivars: this diversity is especially beneficial to hybrid breeding programmes [23, 24, 25]. However, it is difficult to create a new gene pool based directly on RS lines because of their unfavorable agronomic traits, including low yield, poor seed quality [26–27] and self-incompatibility. Other reports have indicated that RS oilseed rape may be crossed with natural oilseed rape to produce semi-RS lines for developing male sterility lines or introgression of new genes for specific breeding procedures, thereby broadening the genetic diversity of this crop [28–29]. *In vitro* androgenesis from the F_1_ hybrids provides the possibility of obtaining large populations of homozygous semi-RS DH lines from which the desired genotypes can easily be selected. In our research, RS plants were used as pollinators in crosses with two double-low restorer lines of winter oilseed rape. From two F_1_ hybrids, 801 semi-RS DH lines were obtained, from which the S1, S3 and S4 lines were selected for the presence of the *Rfo* gene, self-compatibility, low glucosinolate content and the absence of erucic acid [15].

Many reports have indicated that RS lines may be used to develop semi-synthetic oilseed rape, but there are relatively few publications that focus on the yield of RS forms or semi-RS lines under field conditions. Both Zhao et al. [29] and Karim et al. [30] studied the seed yield of a single plant, while Seyis et al. [31], Girke and Becker [24], Girke et al. [8] and Jesske et al. [5] determined the seed yield potential of F_1_ hybrids between male sterile lines and RS oilseed rape based on the results of field experiments. Whereas Gehringer et al. [32] studied yield performance of experimental hybrids resulting from crossing between DH lines (derived from a cross between double-low seed quality winter oilseed rape cultivar and a semi-RS line) and a male sterile tester. However, it should be noted that both the spring oilseed rape hybrids studied by Seyis et al. [31] and the winter oilseed rape hybrids tested by Girke and Becker [24], Girke et al. [8], Jesske et al. [5] and Gehringer [32] did not comply with canola standards.

In our study, the restored hybrids were double-low quality forms. However, their seed yield was not equal to that of the control varieties, except for the M5S3 hybrid, whose yield amounted to 108.6% of the seed yield of open-pollinated cultivar Monolit and 94.4% of the seed yield of hybrid cultivar Arsenal. Jesske et al. [5] also noted that the yield of check hybrid cultivar Visby exceeded all test hybrids. Compared with earlier studies of various RS oilseed rape genotypes [8,29], the heterosis for yield was high and ranged from 10.13% to 90.63% with respect to the mid-parent and from 4.56% to 90.17% with respect to the high parent. Girke et al. [8] observed mid-parent heterosis between male sterile lines (developed from cultivars Falcon and Express) and RS lines ranging from -3.5% to 47.2%, while Zhao et al. [29] described 39.56% mid-parent heterosis and -8.57% high-parent heterosis for hybrids between cultivars and RS lines.

In our study, the highest-yielding hybrid M5S3 resulted from crossing the CMS5 line with semi-RS line S3, although these parents themselves gave some of the lowest yields. All parental components, both maternal sterile CMS lines and paternal semi-RS restorer lines, yielded low in this experiment. The parental lines, especially semi-RS restorer lines, proved very sensitive to cold temperature, which caused severe damage to the plants. Jesske et al. [5] could not estimate heterosis because the paternal RS lines did not survive in the field due to their poor winter hardiness. In our experiment, restorer lines survived cold winter temperatures because their genomes are 50% RS line and 50% natural winter oilseed rape.

The lowest-yielding semi-RS DH line S1 showed the best GCA of the restorer lines used. GCA is an effective tool for the selection of parents based on the performance of their progenies, which are usually the F_1_ plants, but F_2_ and later generations have also been used [33]. A high GCA indicates strong evidence of desirable gene flow from parents to offspring at high intensity and represents information regarding the concentration of predominantly additive genes [34]. One of the main features of the elite parent with a large GCA effect is its high adaptability. A high GCA estimate indicates higher heritability and relatively few environmental effects [33]. However, only one hybrid, i.e. the offspring of the semi-RS line S1, showed high adaptation: hybrid M3S1 yielded significantly more highly than the overall mean. The remaining hybrid progeny of semi-RS S1 line were unstable, as confirmed by GE analysis. In contrast, most of the hybrids pollinated with the semi-RS line S4, which demonstrated the highest yield among restorer lines and gave a highly significant but negative GCA effect, were stable. Nevertheless, a parent that performs well in itself does not necessarily produce better hybrids when used in hybridization programmes [33,35]. Semi-RS restorer line S3, which had a low GCA, produced hybrids that exhibited very different yield levels: two hybrids yielded insignificantly below the overall mean, three hybrids yielded insignificantly above the overall mean; the latter included hybrid M5S3, which yielded the highest of all hybrids. This indicates that one parent of a worst combination could concurrently make the best combination, if the other parent was selected appropriately. As reported by Tiwari [36], parents with the highest GCA effects will not necessarily generate the best specific cross combinations. Good specific combinations for different traits involving good general combiners are expected to produce some useful transgressive segregants, particularly for developing high yielding pure lines, due to an additive effect of gene action. Indeed, there are instances where low × low combiners produce the best combinations. Such behaviour has been attributed to overdominance and epistasis [37]. Maurya and Singh [38] reported that average × average combinations along with high × low combinations produce the best crosses. In this study we obtained similar results. The highest-yielding hybrid M5S3 resulted from a cross between the best CMS combiner line, i.e. CMS5, and a poor combiner semi-RS line, S3. Regardless, neither parental line had a high GCA, and the CMS lines were especially poor combiners. It must be taken into account, however, that the GCA effects of CMS lines were only evaluated on the basis of three cross combinations.

Seed yield is a very complex quantitative trait, whose expression is the result of genotype, environment and the interaction between them [39]. The growing economic importance of oilseed rape means that, globally, the area under cultivation continues to increase. As a result, stable lines, which are characterized by the capacity to adapt to widely different soil and climatic conditions, are considered highly desirable in oilseed rape breeding. Our preliminary analysis showed a significant differentiation between the three locations used in our experiments. GE interaction analysis proved that most hybrids had specific adaptability. The best yielding hybrid (M5S3) was found to be unstable, similar to the control cultivar, Arsenal. Both these genotypes gave the highest yields in Cerekwica, although this was the weakest location in our study, and thus they can be recommended for narrow adaptation under less favorable environments. Among the hybrids demonstrating the highest seed yield stability, the best was M3S1. However, it should be taken into account that field trials were carried out over one season only and the results obtained should therefore be treated as preliminary.

## Conclusions

This study represents the first insights into the application of semi-RS DH lines as paternal components for the development of new hybrid varieties. The introduction of 50% of a resynthesized oilseed rape genotype to natural restorer lines is sufficient to deliver a high heterosis effect. The semi-RS DH line S1, which has a significant GCA for seed yield, can be recommended as a potential parent for inclusion in breeding programmes aimed at developing new hybrid varieties.
